# The Involvement of Thaumatin-Like Proteins in Plant Food Cross-Reactivity: A Multicenter Study Using a Specific Protein Microarray

**DOI:** 10.1371/journal.pone.0044088

**Published:** 2012-09-07

**Authors:** Arantxa Palacín, Luis A. Rivas, Cristina Gómez-Casado, Jacobo Aguirre, Leticia Tordesillas, Joan Bartra, Carlos Blanco, Teresa Carrillo, Javier Cuesta-Herranz, José A. Cumplido Bonny, Enrique Flores, Mar G. García-Alvarez-Eire, Ignacio García-Nuñez, Francisco J. Fernández, Pedro Gamboa, Rosa Muñoz, Rosa Sánchez-Monge, Maria Torres, Susana Varela Losada, Mayte Villalba, Francisco Vega, Victor Parro, Miguel Blanca, Gabriel Salcedo, Araceli Díaz-Perales

**Affiliations:** 1 Centro de Biotecnología y Genómica de Plantas, Campus de Montegancedo, Pozuelo de Alarcón, Madrid, Spain; 2 Departamento de Evolución Molecular, Centro de Astrobiología (INTA-CSIC), Torrejón de Ardoz, Madrid, Spain; 3 Unitat d'Allèrgia, Servei Pneumologia i Allèrgia Respiratòria, Hospital Clínic, Universitat de Barcelona, Institut d'Investigacions Biomèdiques August Pi i Sunyer (IDIBAPS), Centro de Investigaciones Biomédicas en Red de Enfermedades Respiratorias (CIBERES), Barcelona, Spain; 4 Servicio de Alergia, Hospital Universitario de la Princesa, Instituto de Investigación Sanitaria Princesa (IP), Madrid, Spain; 5 Servicio de Alergología, Hospital Universitario de Gran Canaria Dr. Negrín, Las Palmas de Gran Canaria, Spain; 6 Servicio de Alergia, Fundación Jiménez Díaz, Madrid, Spain; 7 Unidad de Alergia, Hospital General Universitario, Alicante, Spain; 8 Unidad de Alergología, Complexo Hospitalario, Ourense, Spain; 9 Laboratorio de Investigación, Fundación IMABIS-Carlos Haya Hospital, Hospital Civil, Málaga, Spain; 10 Servicio de Alergia, Hospital de Basurto, Bilbao, Spain; 11 Departamento de Bioquímica y Biología Molecular I, Facultad de Químicas, Universidad Complutense, Madrid, Spain; 12 Departamento de Biotecnología, ETSI Agrónomos, Universidad Politécnica, Madrid, Spain; University of South Florida, United States of America

## Abstract

Cross-reactivity of plant foods is an important phenomenon in allergy, with geographical variations with respect to the number and prevalence of the allergens involved in this process, whose complexity requires detailed studies. We have addressed the role of thaumatin-like proteins (TLPs) in cross-reactivity between fruit and pollen allergies. A representative panel of 16 purified TLPs was printed onto an allergen microarray. The proteins selected belonged to the sources most frequently associated with peach allergy in representative regions of Spain. Sera from two groups of well characterized patients, one with allergy to Rosaceae fruit (FAG) and another against pollens but tolerant to food-plant allergens (PAG), were obtained from seven geographical areas with different environmental pollen profiles. Cross-reactivity between members of this family was demonstrated by inhibition assays. Only 6 out of 16 purified TLPs showed noticeable allergenic activity in the studied populations. Pru p 2.0201, the peach TLP (41%), chestnut TLP (24%) and plane pollen TLP (22%) proved to be allergens of probable relevance to fruit allergy, being mainly associated with pollen sensitization, and strongly linked to specific geographical areas such as Barcelona, Bilbao, the Canary Islands and Madrid. The patients exhibited >50% positive response to Pru p 2.0201 and to chestnut TLP in these specific areas. Therefore, their recognition patterns were associated with the geographical area, suggesting a role for pollen in the sensitization of these allergens. Finally, the co-sensitizations of patients considering pairs of TLP allergens were analyzed by using the co-sensitization graph associated with an allergen microarray immunoassay. Our data indicate that TLPs are significant allergens in plant food allergy and should be considered when diagnosing and treating pollen-food allergy.

## Introduction

Cross-reactivity is an important problem for the diagnosis and treatment of allergy, and in the daily routine of patients, due to our lack of knowledge about the original sensitization source. Cross-reactivity in plant food allergy is mediated by panallergens belonging to widely distributed protein families. Identifying the patterns of association between different allergen sources from pollen and foods is a priority because of its importance for understanding how allergy is triggered.

*Rosaceae* fruit allergy, represented by peach, is the most prevalent plant ingested allergy in Spain and the south of Europe. It has become notably common in the last years [1]. Pru p 3, the lipid transfer protein (LTP) of peach, is considered to be the main allergen in this fruit, and is recognized by 60–70% of allergic patients [2], [3]. This allergen exhibits cross-reactivity with a wide range of plant foods and some pollen sources, such as mugwort and plane, in a high proportion of patients [3], [4], [5]. However, despite the identification of the major allergen responsible for most peach-associated allergies, we still do not understand the coexistence of cross-reactivity between peach and some fruits or pollens such as grasses.

Recently, members of the thaumatin-like protein (TLP) family have been identified as important allergens in peach fruit [6]. TLPs have also been described as allergens in various fruits, such as apple, cherry, kiwi, olive and banana, and in pollens such as cypress and possibly others. This family is thought to be a panallergen family responsible for cross-reactivity between pollen and fruit, although this is not currently backed up by sufficient experimental evidence [7].

The proteins of the thaumatin-like family have molecular masses of 20–30 kDa, with a very stable three-dimensional structure that is maintained by six disulphide bridges. They have been described as plant defense proteins (PR-5) against pathogen- attacks, especially fungal. Some thaumatins are glycoproteins, and this could account for their allergenic capacity [8].

The involvement of this protein family in cross-reactivity has been determined by *in vitro* techniques, such as ELISA assays, that require large quantities of allergens and serum volume. The onset of microarray techniques with large panels of purified allergens, some of them from the same family, has been a major advance in the diagnosis of allergic diseases [9], [10]. Thus, it is possible to measure simultaneously IgEs, specific to many molecules, using tiny amounts of allergen and sera, thereby enabling a large number of samples to be screened at the reasonable cost. The wealth of information generated by microarrays also demands more powerful analytical strategies to identify associations within the data [11], [12]. For this reason, we have made use of the graph theory to study and visualize the co-sensitization of different sera for TLP allergens. A graph, or network, is composed of nodes and connecting links [13], [14]. These links might be directed or undirected, and weighted or unweighted, depending on the nature of the system under study. In the graphs used in this work, nodes represent allergens, and links (which are undirected and weighted) represent the co-sensitization of sera for pairs of allergens. Recently, these graphs have been used to describe the cross-reactions in an antibody microarray immunoassay in a sandwich format [15], and, in fact, there is an extensive literature about their usefulness for analyzing biological systems [16], [17], [18], [19], [20]. In our particular case, we have focused on developing the potential of graph theory for analyzing TLP microarrays and for applying them in the field.

The principal objective of this study was to establish the role of TLPs in fruit allergy and their putative involvement in cross-reactivities with other foods and/or pollens. For this purpose, 16 members of this family were purified and printed on a protein microarray. The panel of proteins was chosen with respect to the specific features of the sensitization of the population under evaluation [2]. The TLP microarray was tested with the sera from 329 allergic patients from seven regions of Spain, and considering with respect to their different pollen profiles.

## Results

### Purification of TLP Members from Different Foods and Pollens

Peach allergy is usually associated to sensitization to other fruits such as apple, kiwi and banana, and to nuts such as hazelnut, chestnut and walnut [2]. Moreover, over 70% of peach-allergic patients in Spain also suffering from pollinosis, mainly from grasses, mugwort, olive and cypress [2]. Considering these associations, 16 TLPs were purified, from both foods and pollens, according to previously described methods (Table 1). Some of these food-related TLPs had been previously identified as allergens (www.allergen.org, IUIS; www.allergome.com): Act d 2 [8], Cup a 3 [21], Mal d 2 [22], Mus a 4 [23], Pru av 2 [24], Pru p 2.0101 [6], Pru p 2.0201 [6], a wheat TLP [25] and olive TLP [26]. The purified wheat TLP in this study proved to be different from the one associated with baker’s asthma. In this paper, the allergenic activities of the other purified TLPs (such as the proteins from mugwort, birch and plane pollens, and from hazelnut, chestnut, cabbage, lettuce and olive) have been studied for the first time (Table 1). Unfortunately, no TLP from grass pollen could be purified, even though it is one of the most frequently associated with peach allergy.

**Table 1:** Purified proteins included in the TLP microarray.

Protein	Family	Specific/common name	N-terminal or internal peptide sequence	Accession number	Reference
**Act d 2**	TLP	*Actinidia deliciosa*/Kiwi	ATFNI	P83958	[35]
**Cup a 3**	TLP	*Cupressus arizonica*/cypress	VKFDIKNQXRYT	Q69CS2	[21]
**Mal d 2**	TLP	*Malus domestica*/Apple	AKITFTNNXP	Q3BCT8	[22]
**Mus a 4**	TLP	*Musa acuminata*/Banana	ATFEIVNRXSYTVWAAAVPGGGRQLNQ	1Z3Q	[35]
**Pru av 2**	TLP	*Prunus avium*/Cherry	ATISFKNNCP	P50694	This paper
**Pru p 2.0201**	TLP	*Prunus persica*/Peach	R.SVDAPSPWSGR.FAKITFTNKQS	gi190613905	[6]
**Pru p 2.0101**	TLP	*Prunus persica*/Peach	K.ASTCPADINKVCPAPLQVKG AKITFTNK	gi190613911	[7]
**Birch pollen TLP**	TLP	*Betula verrucosa*/Birch	K.NSTFTCSGGPDYVITFCP	Q9FSG7	This paper
**Chestnut TLP**	TLP	*Castanea sativa*/Chestnut	STVIFYNKC	P50699	[38]
**Cabbage TLP**	TLP	*Brassica oleraceae*/Cabbage	ATFEIVNRXS	P02884	This paper
**Hazelnut TLP**	TLP	*Corylus avellana*/Hazelnut	K.NSGFTCSGAFIAAARSNTVWPGTLTGDQKPQLSLTAFELASKA	P83336	This paper
**Lettuce TLP**	TLP	*Lactuca sativa*/Lettuce	ANFNIHNNXP	P83959	This paper
**Mugwort pollen TLP**	TLP	*Artemisia vulgaris*/Mugwort	ATITVXNRXS	Q946Z0	This paper
**Olive TLP**	TLP	*Olea europaea*/Olive	ATFDIVNQCTYTVWAAASPGG	ACZ57583	[39]
**Plane pollen TLP**	TLP	*Platanus acerifolia*/Plane	RCSFTVWPAATPVGGGRQ	P31110	This paper
**Wheat TLP**	TLP	*Triticum aestivum*/Wheat	KASQSVDAPSPWSGRF	P83336	This paper
**Act d 1**	Cystein protease	*Actinidia deliciosa*/Kiwi	LPSYV	P00785	[35]
**Ana c 2**	Cystein protease	*Ananas comosus/*Pineaple	MAEYGRVYKDNDE	BAA21929	Commercial
**Art v 3**	LTP	*Artemisia vulgaris*/Mugwort	ALTXSDV	P0C088	[4]
**Bet v 1**	PR10	*Betula verrucosa/*Birch	ARLFKAFILDGDNL	P15494	Commercial
**Cuc m 2**	Profilin	*Cucumis melon*/Melon	MSWGAYVDDHLMC	AJ565931	[40]
**Pers a 1**	Class I chitinase	*Persea americana*/Avocado	EQHGR	P93680	[41]
**Pho d 2**	Profilin	*Phoenix dactylifera*/Palm	MSWGAYVDEHLMC	AJ417566	Commercial
**Pru p 3**	LTP	*Prunus persica/*Peach	ITCGQE	Q9LED1	[42]

Other allergens were included in the microarray: Pru p 3 (LTP, peach allergen), Art v 3 (LTP, mugwort allergen), Act d 1 (cysteine protease, kiwi allergen), Ana c 2 (pineapple allergen and marker of carbohydrate cross-reactive determinants (CCD)), Bet v 1 (PR10 from birch pollen), Cuc m 2 (melon fruit profilin), Pers a 1 (avocado latex-fruit allergen) and Pho d 2 (palm-pollen profilin).

### Frequency of Recognition of TLPs by Allergic Patients

Seven regions of Spain were chosen on the basis of their characteristic major pollens (Table 2, pollen count average for the previous 10 years), and two groups of patients were included in the study, prospectively among adult population (Table 2): fruit-allergic (FAG) patients and non-food pollen-allergic (PAG) patients. The first group was subdivided into two subgroups depending on whether patients were allergic to pollen (n = 169) or not (n = 43). An additional group of food-tolerant volunteers without pollinosis (n = 35; five per region) was recruited as a negative control.

**Table 2:** Characteristics of the patient sample.

	Total^1^	FAG^2^		PAG^3^
		Pollen	Non-pollen	
Number of patients	329	169	43	117
Sex^4^	112M 217F	57M 112F	15M 28F	40M 77F
Age range	08–62	18–59	08–56	15–62
**Food Allergy Symptoms (%)**				
Rhinitis	61	95	0	87
Asthma	27	34	4	43
Oral allergy syndrome	35	52	54	0
Anaphylactic reaction	14	18	25	0
Urticaria	31	63	28	2
Angio-edema	10	16	14	0
Gastrointestinal	5	9	6	0
Others	2	4	2	0
**SPT (%)** *				
Mugwort pollen	42	50	31	45
Cypress pollen	24	28	13	31
Grass pollen	55	51	27	88
Plane pollen	36	54	23	31
Olive pollen	34	45	18	39
Pellitory pollen	7	5	4	13

1 Total, all patients;

2 FAG, Fruit-allergic patients;

3 PAG, Pollen-allergic food-tolerant patients;

4 M, male; F, female;

* SPTs with purified protein were performed in a selected group of patients of 50 FAG, 20 PAG and 20 non-pollen food-tolerant subjects.

A microarray approach was chosen as the best *in vitro* high-throughput immunological assay to test a large number of proteins and sera, based on the small quantities of protein and sera required. The microarray was constructed by printing TLP proteins onto activated glass slides, following previously published methods [27]. Each protein was recognized by at least one serum, but none of them gave a positive response with sera from the healthy control group (data not shown).

**Figure 1:**
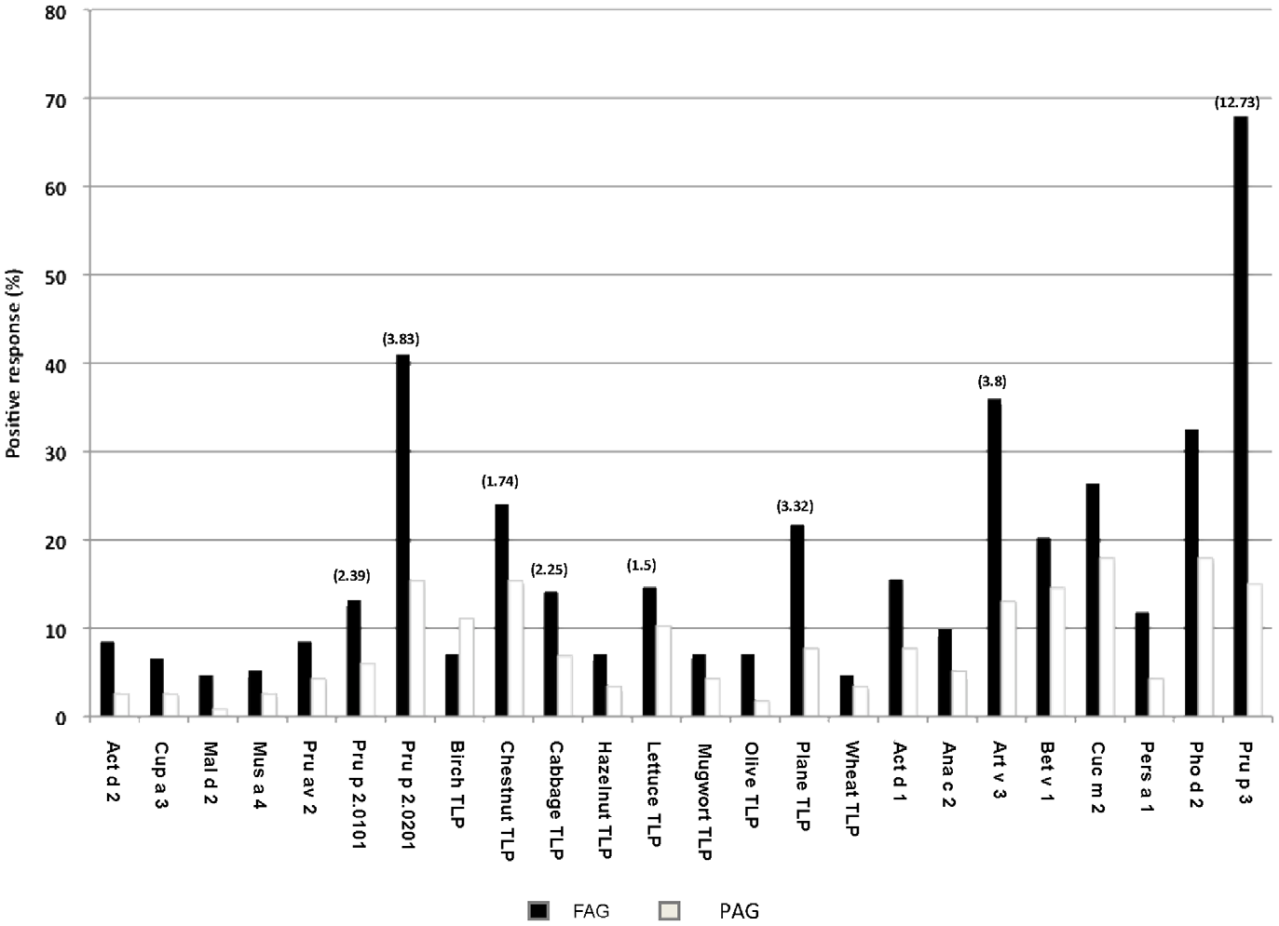
Frequency of sensitization obtained by the TLP microarray using sera from both fruit-allergic (FAG) patients, and non-food pollen allergic (PAG) subjects. Odds ratios are presented in parentheses (95% CI; *p*<0.001).

Generally, TLPs were not highly prevalent in the allergic populations examined in this study (Figure 1). Most of the TLPs (10/16) were recognized by fewer than 10% of the patients. Despite this, they were mainly associated with fruit allergy. The fruit-allergic patients recognized more TLPs than pollinic food-tolerant subjects (2 versus 0.5 on average, respectively). Curiously, patients from Barcelona were significantly different, showing higher polysensitization to these allergens (4 TLPs on average) than did subjects from the other regions (Mann-Whitney U test: *p* = 0.001).

**Figure 2:**
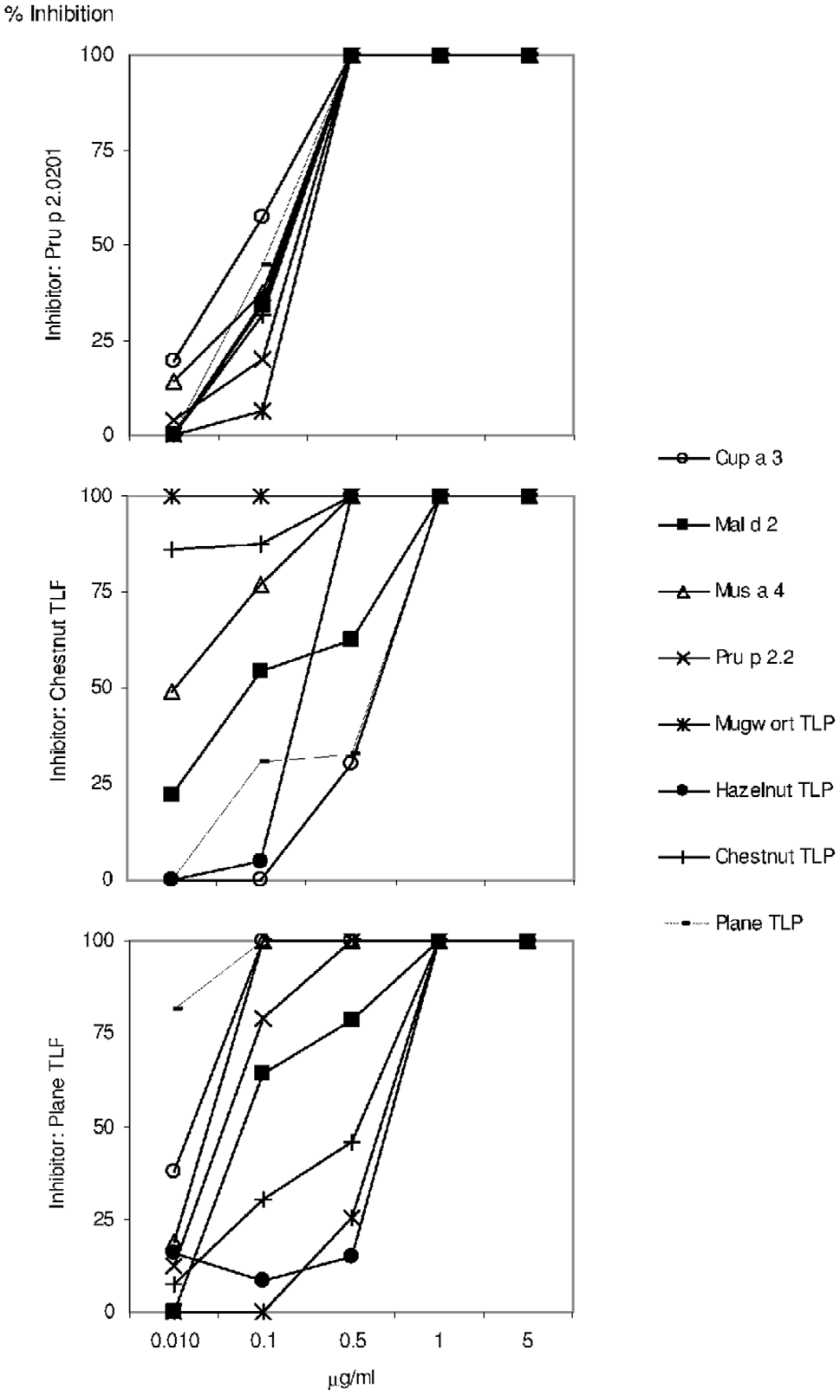
IgE binding inhibition of the TLP microarray, when serum pool (n = 21; three per area) or individual sera were preincubated for 3 h at room temperature with increasing amounts of Pru p 2.0201, and chestnut and plane -pollen TLPs.

Inhibition assays using the peach TLP, Pru p 2.0201, and chestnut and plane pollen TLPs as inhibitors confirmed the cross-reactivity between the members of this family (Figure 2). Pru p 2.0201 seemed to be the starting point for TLP sensitization since it was able to inhibit IgE binding to the other allergens tested (Figure 2).

Fruit allergic group was more likely to have a positive response to Pru p 2.0201(41% *vs.* 15%), chestnut (24% *vs.* 15%) and plane TLP (22% *vs.* 8%) than did patients from the pollen allergic group, who were pollinic subjects but plant-food tolerant attending to microarray results (χ^2^ = 0.002, 0.017 and 0.003, respectively). Individuals affected by fruit and pollen allergy more frequently recognized TLPs than did those without respiratory allergy. In the case of Pru p 2.0201, 41% of patients allergic to pollen and fruit -had a positive response in contrast to 33% of individuals in the same group without pollen allergy. Pru p 2.0201 was the most prevalent allergen, being recognized by 32% of all the patients (FAG+PAG) studied. A close association between sensitization to Pru p 2.0201 and to Pru p 3 was observed (χ^2^ = 0.005).

### Response Pattern by Geographical Area

**Figure 3:**
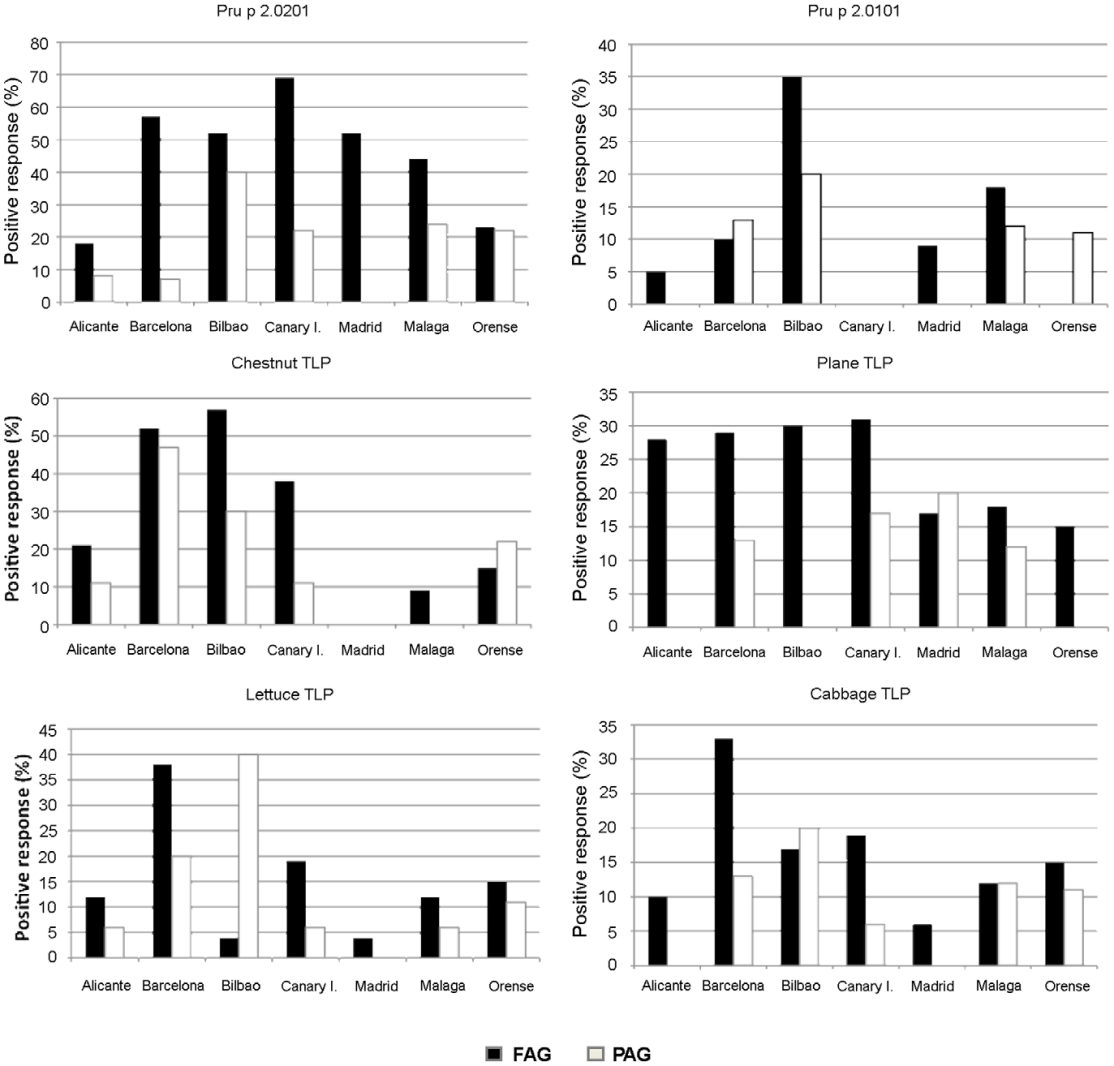
Frequency of sensitization in the different geographical areas using the homemade microarray and sera from fruit-allergic (FAG) patients, and non-food pollen-allergic (PAG) subjects. Only TLPs with more than 10% positive response were represented: Pru p 2.0201, Pru p 2.0101, chestnut, plane, lettuce and cabbage TLPs.

To study the differences in the TLPs recognition in patients with fruit allergy, we selected patients from 7 geographical areas with different pollen profiles (Table 3). The analysis of prevalence by geographical area revealed some specific features about recognition frequencies in fruit allergy group of patients (Figure 3).

**Table 3:** Pollen counts (grains/m^3^ of air) of the regions included in the study.

Region Pollen*	Alicante (2001–2011)	Barcelona (2001–2011)	Bilbao (2001–2011)	Canary I. (2008–2011)	Madrid (2001–2011)	Málaga (1995–1999)	Ourense (1996–1999)
**Grass**	–	–	–	–	200	–	–
**Birch**	–	–	100	–	–	–	120
**Plane**	–	1800	–	–	1050	225	–
**Oak**	–	375	330	–	520	–	100
**Pine**	375	200	620	100	160	–	130
**Cypress**	175	525	–	–	200	250	470
**Mugwort**	–	–	–	150	–	–	–
**Pellitory**	–	–	–	110	–	–	–
**Olive**	355	125	–	–	473	1250	–
**Palm**	220	–		–	–	–	–

Data were obtained as the average of the previous n years, from the Comité de Aerobiología-SEAIC (http://www.polenes.com/concentraciones.html) and from PIA-Punto de información de Aerobiología-UAB (http://lap.uab.cat/aerobiologia/).

* Average of pollen counts (grains/m^3^). The time period is indicated in brackets.

Significant differences were observed in response to Pru p 2.0201 (χ^2^<0.0001), ranging from 18–23% in patients from Alicante/Ourense respectively, to 70% in those from the Canary Islands. These results contrasted with the prevalences of Pru p 2.0101, the other peach TLP, which was less than 10% in every region, except for the FAG patients from Bilbao (35%; χ^2^ = 0.012) and from Malaga (18%; χ^2^ = 0.120). In fact the frequencies of both peach TLPs (Pru p 2.0201 and Pru p 2.0101) were especially high in patients with pollen sensitization but with plant food tolerance (PAG) from Bilbao. The same pattern was observed for the lettuce TLP. This allergen was recognized by 40% of PAG subjects from Bilbao, although almost no recognition was detected in FAG patients (<5%). This raises the possibility that respiratory allergies affect the recognition of TLP allergens. However, this suggestion needs more evidences.

Chestnut TLP and plane TLP were found to be important in fruit sensitizations. The former proved to be a significant allergen in fruit-allergic patients from Barcelona and Bilbao, with recognition frequencies of more than 50%, and from the Canary Islands, with almost 40% recognition. In Barcelona, 48% of pollen-allergic patients with plant-food tolerance had a positive response. In all other study areas, limited recognition was observed. The case of the plane TLP was quite different. The most striking recognition level (around 30%) was exhibited by fruit-allergic patients from Alicante, Barcelona, Bilbao and the Canary Islands. This allergen was the only pollen TLP that appeared to be important in fruit allergy.

### Graph-based Analysis of the TLP Microarray Immunoassay

**Figure 4:**
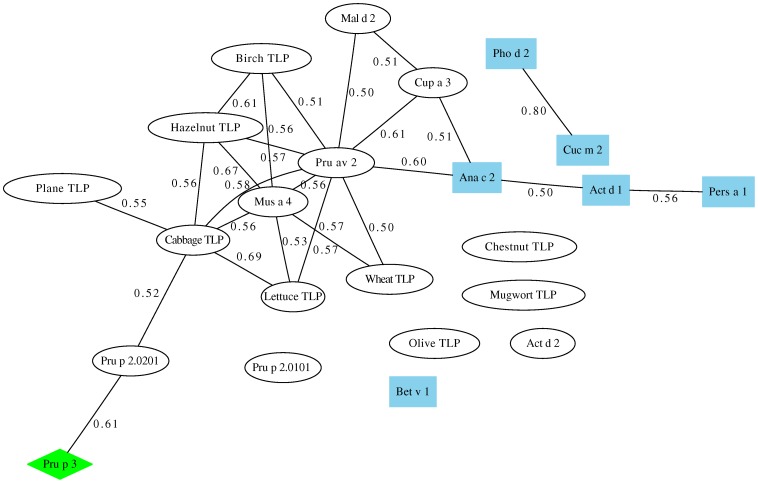
Co-sensitization graph of TLP allergens. Each node represents one allergen (TLP as white ellipses, non-TLP allergens as blue square nodes, and LTP-allergen Pru p 3 as a green diamond) and the links represent co-sensitization of one or more sera for the linked allergens. The weight of each link, ranging between 0 and 1, measures the degree of co-sensitization. For the sake of clarity, only the 25 links of weight greater than 0.50 out of the total 253 existing links were plotted.

We made use of the graph theory for two main reasons. Firstly, it is a simple and useful way of representing TLP-microarray immunoassay data, and secondly, because we wanted to gain insight into the co-sensitization patterns of the TLP allergens in the selected population. The steps for building the co-sensitization graph associated with our TLP microarray immunoassay (Figure 4) are explained in detail in the Materials and Methods. In short, two allergens are connected by a link in the graph if at least one serum gave a positive reaction to both allergens, and the weight of such a link is a measure of the degree of similarity or correlation between the sera that reacted positively to each of the allergens -that is, such weight depends on whether these two sets have common elements or not-. Therefore, the maximum weight 1 was assigned when both allergens were recognized by exactly the same group of reacting sera (irrespective of the size of the group).

The co-sensitization graph showed to be totally connected (i.e., all allergens were connected with all other allergens), meaning at least one serum reacted positively to any pair of allergens, or in other words, that there were no incompatible pairs of co-sensitizations. Therefore, there are (23×22)/2 = 253 links in the graph. However in order to clarify the interpretation of the results only the 25 links with weights greater than 0.50 were plotted in Figure 4.

The TLP allergens connected with the highest weights were cabbage-TLP/Lettuce TLP (0.69) and hazelnut-TLP/Mus a 4 (0.67). By contrast, the strongest link connected the non-TLP allergens Cuc m 2 and Pho d 2 (0.80), both belonging to the family of, profilins. The lowest co-sensitizations were found between Mal d 2/Pru p 2.0201 (0.12), wheat-TLP/Pru p 2.0101 (0.14), and wheat-TLP/Mal d 2 (0.16).

The average weights (Table 4) were a measure of the degree of co-sensitization that an allergen showed with the rest of the allergens in the graph. Pru av 2, cabbage TLP and Lettuce TLP had the highest average weights (all above 0.4), meaning that their co-sensitizations with the rest of the network are especially relevant. The case of the non-TLP allergens is remarkable, as they have relatively large average weights (especially Ana c 2 (CCD marker) with a value of 0.38), even when they were used as control proteins in the experiments. Finally, we should mention the case of olive TLP and Pru p 3, the allergens linked with the lowest average weight (both 0.29).

**Table 4:** Average weight of allergens included in the co-sensitization graph.

Protein	Family	Average weight
Pru av 2	TLP	0.43
Cabbage TLP	TLP	0.42
Lettuce TLP	TLP	0.40
Ana c 2	Cysteine proteinase	0.38
Plane pollen TLP	TLP	0.37
Cup a 3	TLP	0.36
Pers a 1	Class I chitinase	0.35
Act d 1	Cysteine protease	0.35
Mus a 4	TLP	0.34
Hazelnut TLP	TLP	0.34
Pho d 2	Profilin	0.34
Chestnut TLP	TLP	0.33
Birch pollen TLP	TLP	0.33
Cuc m 2	Profilin	0.33
Mal d 2	TLP	0.33
Pru p 2.0201	TLP	0.32
Mugwort pollen TLP	TLP	0.32
Bet v 1	PR10	0.31
Wheat TLP	TLP	0.30
Act d 2	TLP	0.30
Pru p 2.0101	TLP	0.30
Pru p 3	LTP	0.29
Olive TLP	TLP	0.29

## Discussion

The study of cross-reactivity in allergy using protein-microarray strategies is a powerful method with advantages over other immunological approaches [9], [11]. A large number of allergens and sera from many patients can be tested in the same assay, with the additional advantages of lower costs and the more rapid processing of samples [12], [28], [29], [33].

In Spain, fruit allergy is clearly associated with LTP sensitization, especially to Pru p 3 [2], [3], the peach allergen. However, LTPs are not the only proteins involved in fruit/food sensitization. Other families of allergens, such as profilins [30], [31], [32], have been described as being important in the development of food allergy in this area.

Members of the TLP family can have a role as allergens in a wide panel of plant food and several pollens, although there is little experimental evidence in plant foods and/or pollen cross-reactivities [6], [7]. In this paper, we have tried to establish the role of this protein family in plant food allergy and in cross-reactivity between foods and pollens. A large number of patients were selected from different Spanish regions and a representative panel of TLPs based on the most frequent sensitizations associated with peach allergy [2] was printed in a protein microarray.

In order to study the association between the different allergens in a visual and intuitive manner, we have presented our results in a co-sensitization graph. In this, the nodes represent the different allergens, and the weight of the link that connects two allergens measures whether the sera that reacted positively to one allergen also gave a positive reaction to the other. Therefore, the weight of each link gives us a quantitative impression of the co-sensitization of sera for that pair of allergens.

The analysis of the recognition profile revealed that fruit-allergic (FAG, independent of their respiratory sensitization) subjects showed a strong positive response to several TLPs, although this response tended to be more frequent in patients with pollinosis. However, people with isolated pollen allergy (PAG) showed no predominant recognition pattern. They had a low level of positive responses to TLPs.

Only six of the 16 TLPs studied (the two peach, chestnut, lettuce, cabbage and plane TLPs) yielded recognition frequencies greater than 10%. Of particular interest is the significantly small number of responders we obtained with Mal d 2 and Pru av 2 (5%), which are both important allergens in central and northern Europe [7], [34]. This low response may be related to the allergic profile of the patients included in this work, who were mainly sensitized to peach.

The peach TLP, Pru p 2.0201, gave more than 40% positive responses in fruit-allergic patients, with values of up to 50% in areas such as Barcelona, Bilbao, the Canary Islands and Madrid. This protein seems to act as the gateway for sensitizing members of this family. An unexpected pattern of association between the two LTP allergens (Pru p 3 and Art v 3) and the TLP Pru p 2.0201 was observed. The recognition of Pru p 3 and Pru p 2.0201 was closely associated (χ^2^ = 0.005), and the weight of its co-sensitization link was 0.61.

While most of the patients sensitized to this peach TLP (Pru p 2.0201) showed a positive response to Pru p 3, the opposite was observed for Pru p 3 to the other peach TLP, Pru p 2.0101, whose weight was very low (0.35). This allergen had a low frequency of positive responses (<15%), with the exception of FAG patients from Bilbao (35%). Both peach TLPs are linked with a low co-sensitization weight (0.40), although both of them share more than 94% of amino acid identity, with only eight different residues [7], [30]. Thus, a different mode of sensitization may operate, which would be interesting to study in more detail.

The chestnut TLP yielded a higher positive response in patients from Barcelona and Bilbao, mainly in the FAG group, but also in the PAG group from Barcelona. The plane TLP was the only pollen included in this study that was associated with fruit allergy, especially in areas such as Alicante, Barcelona, Bilbao and Madrid. The other pollen TLPs, like mugwort and birch, had low responses in our population, although patients sensitized to these were included.

As mentioned above, TLPs were related to fruit allergy predominantly with pollen, but they also had sensitization profiles associated with the study geographical areas. Patients from Barcelona, Bilbao and the Canary Islands showed a higher positive response to these allergens than subjects from the other areas. Alicante and Ourense were situated at the other end of the peninsula. Patients from these areas had the lowest frequency of TLP recognition (around 20%, with the exception of plane TLP in Alicante). The patterns of TLP recognition, related to the different geographical areas, suggest a possible influence of local pollen in TLP sensitization.

Studies with large protein and serum panels are needed to clarify the role of cross-reactive allergens, and provide immunological evidence to clinical observations. In this way we can begin to detail the actors involved. In this work, we have shown that few members of the thaumatin family have an important role in fruit/pollen allergy in the studied areas, even if they can act as modifiers of sensitization profiles.

## Materials and Methods

### Selection and Purification of Allergens

Considering the allergies most frequently associated with peach sensitization in Spain, 16 TLPs were purified from foods and pollen relevant to the population under study, based on previously published methods [6], [8], [23], [35] (Table 1).

Other allergens were included in the microarray: Pru p 3 (LTP, peach allergen), Art v 3 (LTP, mugwort allergen), Act d 1 (cysteine protease, kiwi allergen), Ana c 2 (pineapple allergen and marker of carbohydrate cross-reactive determinants (CCD)), Bet v 1 (PR10 from birch pollen), Cuc m 2 (melon fruit profilin), Pers a 1 (avocado latex-fruit allergen) and Pho d 2 (palm-pollen profilin).

All purified proteins were identified by trypsin peptide- and/or N-terminal amino acid-sequencing and mass spectrometry (MALDI-TOF).

### Characteristics of the Regions Under Study

Seven regions of Spain were chosen on the basis of their characteristic major pollens (Table 3, pollen count average for the previous 10 years). In Ourense and Bilbao, in the north of Spain, the most abundant pollens are from pine and oak, but the most distinctive feature is that birch pollen can be found in both regions; Barcelona, on the Mediterranean coast, is characterized by the presence of plane, oak and cypress pollen; Madrid, in the central region, has a high predominance of plane, oak and olive pollen; Málaga, in the south, has a high level of olive pollen; Alicante (Elche) is dominated by pine, olive and palm pollen; and finally, the Canary Islands, in the Atlantic Ocean, are characterized by mugwort and pellitory pollen.

### Characteristics of the Allergic Population Included

Two groups of patients were included in the study, prospectively among adult population (Table 2): fruit-allergic (FAG) patients and non-food pollen-allergic (PAG) patients. Criteria for inclusion in the FAG group (n = 212 patients) were: a consistent history of adverse reaction to fruit, indicative of IgE-mediated allergy, giving positive results to the skin-prick test and open food challenge, following the diagnostic algorithm recommended by official allergy academies [36], [37]. Patients suffering severe systemic reactions to peach, and those with typical, recent and repeated reactions who had positive skin-prick tests did not undergo an oral challenge test to diagnose plant food. This group was divided into two subgroups depending on whether patients were allergic to pollen (n = 169; following the same criteria as for PAG, described below) or not (n = 43).

Criteria for inclusion in the PAG group (117 patients) were: a compatible clinical history of pollinosis confirmed by positive skin-prick tests to pollen allergens, but without any symptoms of plant food allergy and with a negative response to food extracts by SPTs. These patients showed mainly positive responses to mugwort, olive and grass pollen.

An additional group of food-tolerant volunteers without pollinosis (n = 35; five per region) was recruited as a negative control. Most of them (n = 27) were atopic, suffered from dust mite and animal dander allergies.

SPT responses were performed following EAACI recommendations [36]. The Ethics Committee of each hospital approved the study: the Ethic Committee of Hospital Clinic de Barcelona; the Ethic Committee of Hospital Universitario de la Princesa; the Ethic Committee of Hospital Universitario de Gran Canaria Dr. Negrín; the Ethic Committee of Fundación Jiménez Díaz; the Hospital General de Alicante; the Ethic Committee of Complexo Hospitalario de Ourense the Ethic Committee of Hospital Civil, Málaga; the Ethic Committee of Hospital de Basurto, Bilbao; the Ethic Committee of Universidad Politécnica de Madrid (Spain). Patients and control volunteers also gave their written informed consent to their participation.

### PrintingProduction of Allergen Microarray and Immunoassays

Purified proteins were printed (0.25 mg/ml and 0.125 mg/ml in 1X Protein Binding Buffer (Whatman, USA) containing 0.02% Tween 20) on epoxy-activated glass slides (TeleChem International, Sunnyvale, CA, USA) with 16 microarrays per slide, using a MicroGrid II TAS microarrayer (BioRobotics, Genomic Solutions, US). Several protein concentrations (1, 0.75, 0.5, 0.25 and 0.125 mg/ml) were tested and those that resolved the best were chosen (data not shown). Labeled pre-immune antibody was spotted as a guide dot to support automatic image analysis. Gaskets (TeleChem International, Sunnyvale, CA, USA) were attached to the slides to create a barrier between the 16 microarrays and sealed to prevent evaporation. Each microarray well was incubated for 1 hour at room temperature with blocking solution (Sigma, St. Louis, CO, USA), then incubated overnight with 80 µL of serum at 4°C. To detect bound IgE antibodies, the slides were incubated for 1 hour at room temperature with anti-human IgE labeled with PE-DY 647 (Thermo Scientific, Rockford, IL, USA) diluted 1∶100. As a blank control, one microarray well per slide was always incubated solely with PBS (Sigma, St. Louis, CO, USA) instead of serum and, after washing, incubated with the fluorescence secondary antibody. PBS containing 0.1% Tween 20 was used as washing solution. Three points from the same sample were included in each microarray, and three replicates of each assay were performed (Pearson correlation = 0.83; *p*<0.0001).

The inhibition assays were performed in the same way but using sera preincubated with different quantities (5, 1, 0.5, 0.250, 0.125 mg/mL) of inhibitors (Pru p 2.0201, chestnut and plane-pollen TLPs).

Spots with obvious defects and those replicate spots having a signal-to-noise ratio less than 3, as measured by GenePix™ software (Genomics Solutions, US), were removed from the analysis. Only those allergen spots in which at least two of the three replicates fulfilled the analytical criteria were considered for quantification. The IgE binding of each allergen spot was calculated as the final fluorescence intensity, obtained by subtracting the local background *B* from the observed value, measured by GenePix™ software and then the fluorescence intensity from the blank control by applying the next equation: *I*  =  (F_645_–*B*)_sample_ – (F_645_–*B*)_blank_. Fluorescence intensity levels >200 units were considered to be positive (highest value of mean +3×SD of negative control spots, those containing only blocking solution).

### Graph-based Analytical Study of the Associations between TLPs. Building up the Co-sensitization Graph Associated with a TLP Microarray Immunoassay

A weighted and undirected co-sensitization graph 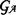 is associated with a TLP microarray immunoassay for studying the co-sensitization between TLPs in the following manner. We define the elements of the matrix of fluorescence intensities ***I*** as the fluorescences *I_ij_* obtained when the serum IgE *i* binds to the allergen *j* as explained above. Therefore, each column of ***I*** represents the average of two different microarray immunoassays in which the same patient serum is incubated on the TLP microarray and revealed with fluorescently labeled anti-human IgE. We call ***B*** the matrix defined by *B_ij_*  = 1 when *A_ij_ >0* and *B_ij_*  = 0 otherwise.

Bipartite graphs are those with nodes of two or more different natures, where links only connect nodes of different type. The graph 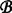 associated with matrix ***B*** is of this kind. Nodes of type S represent the *N_S_*  = 329 sera and nodes of type A represent the *N_A_*  = 23 allergens (16 TLPs, 6 non-LTP allergens, and Pru p 3). Two nodes *i* (serum) and *j* (allergen) are connected in the bipartite graph 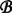 if *B_ij_*  = 1, which means that subject *i* has shown a positive allergic reaction to the allergen *j*.

The bipartite graph 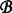 (not shown in this paper) can be projected into two smaller graphs 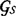 and 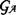, each of them with nodes of only one type (sera and allergens, respectively). The present study focuses on the projected allergen graph 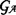, and we have called it the co-sensitization graph (the construction of the projected serum graph 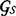 would be similar). The links in graph 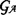 connect two allergens *m* and *n* if, in the original bipartite graph 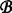, these two allergens were connected to one or more common sera. The weight *w_m,n_* of such a link between *m* and *n* takes values from 0 to 1 and measures the *similarity* between the neighbours of allergens *m* and *n* in the bipartite graph. We have used the definition of the cosine distance between two vectors to calculate *w_m,n_*,:
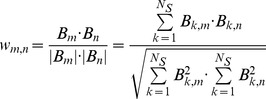
where *B_m_* and *B_n_* are the *m-* and *n-*columns of matrix *B* respectively. Note that the weight *w_m,n_* is zero, and therefore there is no link between allergens *m* and *n*, when *B_m_* and *B_n_* have no common elements (that is, when not even one serum has a positive reaction to both allergens *m* and *n*), while *w_m,n_* reaches its maximum value of one when both vectors are identical (that is, when allergens *m* and *n* are recognized by the same group of reacting sera).

Finally, an important quantity is the average weight of a node, which is calculated as.
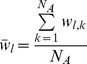



The average weight of an allergen *l* measures the average robustness of the co-sensitizations between *l* and the rest of the allergens represented in the graph.

### Statistical Analysis

Fluorescence levels obtained from each patient’s serum were analyzed by contingency tests. Pearson correlation coefficients were considered as a measure of reproducibility. Associations of frequencies were assessed with the Chi-square (χ2) test. Differences in the quantitative variables were analyzed by the Mann-Whitney U test. Significance was concluded for values of *p*<0.05.
